# Comprehensive analysis of lncRNAs N^6^-methyladenosine modification in colorectal cancer

**DOI:** 10.18632/aging.202383

**Published:** 2021-01-20

**Authors:** Luo Zuo, Hui Su, Qiao Zhang, Wei-yu Wu, Yan Zeng, Xue-mei Li, Jing Xiong, Lan-fang Chen, Yan Zhou

**Affiliations:** 1 The Gastroenterology Tumor and Microenvironment Laboratory, Department of Gastroenterology, The First Affiliated Hospital of Chengdu Medical College, Chengdu Medical College, Chengdu 610041, Sichuan, PR China

**Keywords:** lncRNAs, m6A, colorectal cancer

## Abstract

Background: Long non-coding RNAs (lncRNAs) and their N6-methyladenosine (M6A) modifications are involved in cancer occurrence and development.

Methods: lncRNA M6A modification in colorectal cancer (CRC) was comprehensively analyzed for the first time.

Results: M6A levels of lnRNAs in CRC tissues were higher than those in tumor-adjacent normal tissues. A total of 8,332 M6A peaks were detected in 6,690 lncRNAs in CRC tissues. Approximately 91% of the modified lncRNAs had unique M6A modification peaks. A total of 383 lncRNAs were differentially methylated in CRC, of which 48.24% had a length of 1-1,000 bp. Most of these were located on chromosomes 1, 2, 7, 11, 16 and 19; 42.3% were within a sense-overlapping exon. RNA sequencing identified 163 differentially expressed lncRNAs in CRC. GO and KEGG analyses revealed that genes near differentially-methylated or -expressed lncRNAs were associated with CRC occurrence and development. Methylation was positively correlated with lncRNA expression levels in CRC and tumor-adjacent normal tissues. More unmethylated than M6A methylated lncRNA molecules were detected. A competing endogenous RNA (ceRNA) and lncRNA-mRNA expression-regulation network revealed a regulatory relationship between lncRNAs, microRNAs (miRNAs), and mRNAs.

Conclusions: The findings may help improve our understanding of lncRNA function in colorectal cancer.

## INTRODUCTION

Colorectal cancer (CRC) is the second most common cause of cancer-related death [1]. Epidemiological analysis has revealed that over 50% of patients with CRC have liver metastasis [2]. Although the treatment of CRC has been continuously optimized as medical technology has advanced, there is still no effective treatment for patients with advanced metastatic CRC. One of the basic processes during CRC occurrence and development is the accumulation of various genetic and epigenetic changes in colonic epithelial cells [3, 4]. Epigenetic modifications, including DNA methylation, histone modification, nucleosome remodeling, and RNA modification [5], regulate cell self-renewal, differentiation, invasion, and apoptosis by modulating gene expression [6].

RNA can be modified by N6-methyladenosine (M6A), 5-methylcytosine (m (5) C), and pseudouridine [7]. M6A refers to an adenosine at the N6 position that has a methyl group attached to it and is the most abundant and evolutionarily conserved RNA modification; M6A can be found in most organisms, from bacteria to mammals [8, 9]. Abnormal M6A modification is closely related to the occurrence of a variety of tumors, such as hepatocellular carcinoma, breast cancer, lung cancer, and CRC [10–13]. Abnormal M6A modification has been suggested to contribute to tumor stem cell self-renewal and promote cancer cell proliferation [14]. However, M6A has also been found to affect tumor progression by regulating the expression of oncogenes or tumor suppressor genes, suggesting a dual regulatory role for M6A in cancer [15, 16].

Non-coding RNAs (ncRNAs) are non-protein coding RNAs that primarily include small ncRNAs and long non-coding RNAs (lncRNAs). Although lncRNAs encode almost no protein, they can regulate gene expression through RNA interference, gene co-inhibition, gene silencing, gene imprinting, and DNA demethylation [17, 18]. LncRNAs also participate in various molecular biological processes. For example, lncRNAs play a key regulatory role in the occurrence and development of many diseases, including CRC [19]. *LINC00858* plays a tumor-promoting role in CRC by upregulating *HNF4α* and down-regulating *WNK2* [20]. Additionally, the lncRNA *HITT* inhibits *HIF-1* mRNA expression and inhibits angiogenesis and tumor growth *in vivo* by interfering with the translation of HIF-1 α [21]. Increased expression of the lncRNA *ITIH4-AS1* mediated by the downregulation of the Re1-silencing transcription factor can promote the growth and metastasis of CRC cells by activating the JAK/STAT3 signaling pathway [22]. Therefore, it is essential to investigate lncRNAs that are differentially expressed between CRC tissue and tumor-adjacent normal tissues to elucidate the mechanisms by which lncRNAs influence the growth, metastasis, and invasion of CRC cells.

M6A methylated lncRNAs also have important regulatory functions in a variety of biological and pathological processes. In osteosarcoma cells, M6A modification promotes cellular proliferation and growth by upregulating lncRNA plasmacytoma variant translocation 1 [23]. M6A modification of *LincRNA1281* affects the differentiation potential of mouse embryonic stem cells [24]. In addition, M6A modification regulates CRC proliferation and metastasis by affecting the expression of the lncRNA XIST [25]. However, the abnormal expression and M6A methylation of lncRNAs in CRC cells and their therapeutic applications remain unclear. Therefore, the establishment of a transcriptome map of the expression and M6A modification of lncRNAs in CRC is of great significance to understand the mechanisms by which lncRNAs affect the growth, metastasis, and invasion of CRC cells.

## RESULTS

### General features of M6A methylation of lncRNAs in CRC and tumor-adjacent normal tissues

**Figure 1:**
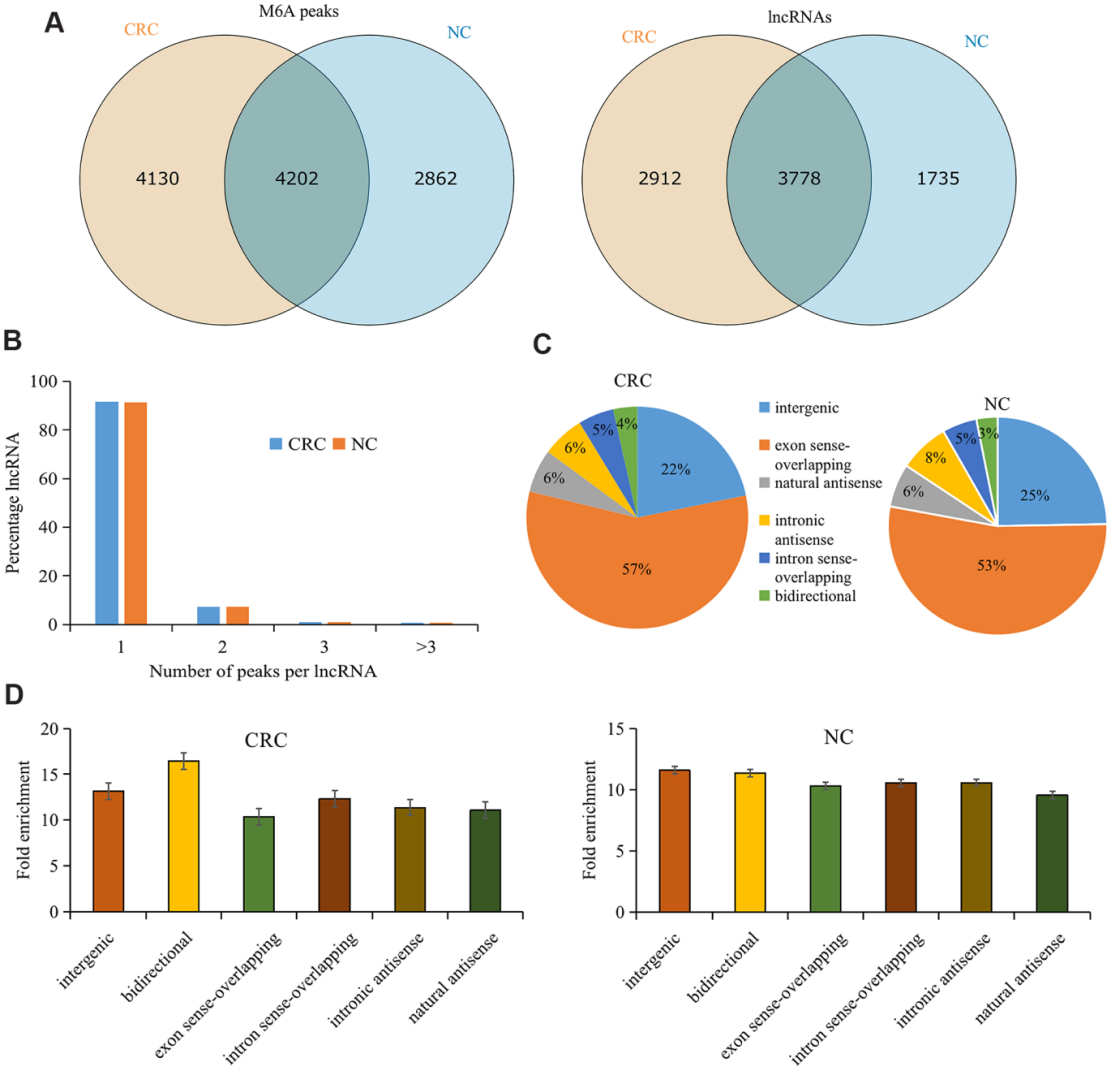
**Overview of M6A methylation within lncRNAs in CRC and tumor-adjacent normal tissues.** (**A**) Venn diagram showing the numbers of cancer-unique, normal-unique, and common M6A peaks within lncRNAs in the two groups. (**B**) Proportions of lncRNAs harboring different numbers of M6A peaks in the two groups. (**C**) Pie charts showing the percentages of the positional relationship between M6A methylated lncRNAs and mRNAs in the two groups. (**D**) Distributions of fold enrichment of M6A peaks in six categories. Colorectal cancer, CRC; N6-methyladenosine, M6A; tumor-adjacent normal tissues, NC.

There were 8,332 M6A peaks detected within 6,690 lncRNAs in CRC tissues, while 7, 064 M6A peaks were detected within 5,513 lncRNAs in the tumor-adjacent normal tissues. Among them, 4,202 peaks (accounting for only 37.5% of all peaks in the two groups) were shared between CRC and tumor-adjacent normal tissues (Figure 1A). The low percentage of shared M6A peaks within lncRNAs indicates a difference in the M6A pattern between the two groups.

Analyzing the distribution of M6A peaks in each lncRNA revealed no significant difference between the two groups. Approximately 91% of the modified lncRNAs had unique M6A peaks, and approximately 7% of the modified lncRNAs had two M6A peaks (Figure 1B), which is consistent with the proportions previously reported for mRNAs [26].

To further understand the M6A methylated lncRNAs in CRC and tumor-adjacent normal tissues, they were divided into the following six categories based on the positional relationship between the M6A methylated lncRNA and mRNA: bidirectional, exon sense overlap, intron sense overlap, intron antisense, natural antisense, and intergenic. Our results revealed that the majority of lncRNAs contained M6A sites with exon sense overlap (CRC tissues accounted for 57.02% and tumor-adjacent normal tissues accounted for 52.9% of such lncRNAs), and most lncRNAs contained intergenic M6A sites (CRC tissues accounted for 21.8% and tumor-adjacent normal tissues accounted for 24.6% of such lncRNAs). Only approximately 3.5% of the methylation sites were in the bidirectional group (Figure 1C). Furthermore, M6A peaks demonstrated a higher fold enrichment in the bidirectional group in CRC tissues. However, in tumor-adjacent normal tissues, the natural antisense group harbored peaks with higher fold enrichment (Figure 1D), indicating differential M6A patterns in CRC and tumor-adjacent normal tissues.

### Length and distribution of differentially methylated lncRNAs

**Figure 2:**
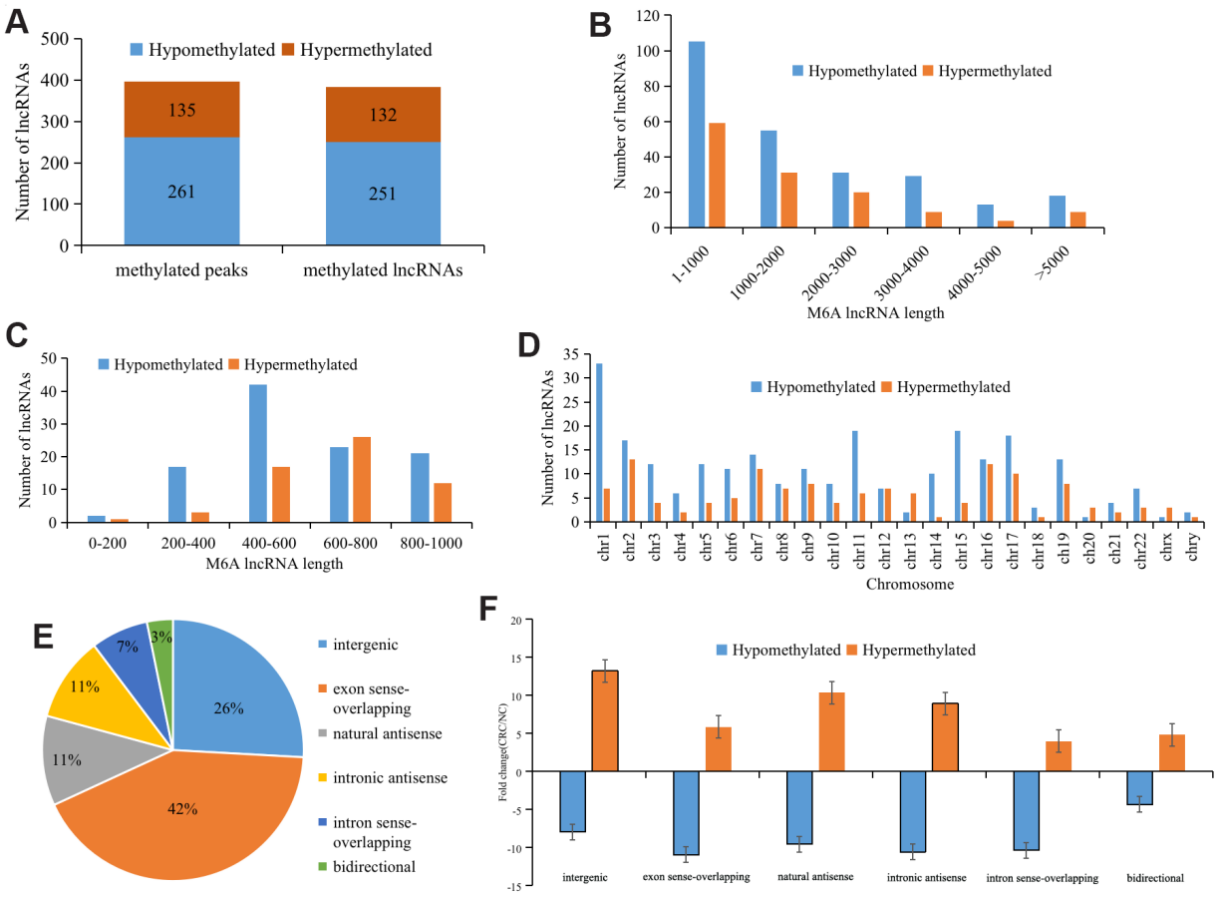
**Distribution of lncRNAs with differential M6A modification.** (**A**) General numbers of differentially methylated peaks and associated lncRNAs. (**B**) Length of differentially M6A methylated lncRNAs. (**C**) and (**D**) Distribution of differentially expressed M6A lncRNAs on chromosomes. (**E**) Positional relationship between differentially M6A methylated lncRNAs and mRNAs. (**F**) Histogram showing the mean fold change of differentially methylated M6A peaks. Error bars represent the standard error of the mean.

We identified 396 differentially methylated M6A peaks, of which 65.9% (261/396) were significantly hypomethylated and hypermethylated, accounting for 34.1% (135/396) (Figure 2A). The 396 differentially methylated M6A sites were located across 383 lncRNAs; of these lncRNAs, 65.5% (251/383) were hypomethylated and 34.5% (132/383) were hypermethylated (Figure 2A). The top ten hypermethylated or hypomethylated lncRNAs are shown in Table 1.

**Table 1:** Top ten hypermethylated or hypomethylated lncRNAs.

**chrom**	**txStart**	**txEnd**	**GeneName**	**Foldchange**	**regulation**
chr21	40400925	40401053	AF064858.10	1135.5	hypermethylated
chr2	43256052	43256660	LOC102723854	944	hypermethylated
chr10	13246218	13246526	MCM10	650.5	hypermethylated
chr8	145580221	145580373	FBXL6	529.3	hypermethylated
chr1	31989461	31989846	LINC01226	503.3	hypermethylated
chr12	114204879	114204900	LINC01234	468.7	hypermethylated
chr11	61521500	61521917	DKFZP434K028	460	hypermethylated
chr15	28594381	28594740	RP11-483E23.2	451.3	hypermethylated
chr3	101679229	101679575	RP11-221J22.2	330.1	hypermethylated
chr3	186454327	186454439	RP11-573D15.8	295.4	hypermethylated
chr14	94406121	94406460	ASB2	138.3	hypomethylated
chr21	44188481	44188522	PDE9A	99.7	hypomethylated
chr11	68781258	68781260	MRGPRF-AS1	77.5	hypomethylated
chr16	1277701	1277790	TPSB2	72.8	hypomethylated
chr16	10273781	10274120	GRIN2A	52.9	hypomethylated
chr2	98967381	98967603	AC092675.4	50.9	hypomethylated
chr4	144465241	144465509	SMARCA5	50.2	hypomethylated
chr14	103606011	103606120	RP11-736N17.4	46.1	hypomethylated
chr15	57982841	57983080	GCOM1	39.2	hypomethylated
chr10	7659681	7659940	ITIH5	32.9	hypomethylated

Analyzing the length of differentially M6A methylated lncRNAs revealed no significant difference between those that were hypermethylated and those that were hypomethylated, and the length of the methylated lncRNAs was primarily 1-1,000 bp (48.24%) (Figure 2B). Further analysis of the methylated lncRNAs with a length of 1-1,000 bp indicated that 44% of the hypermethylated lncRNAs were 600-800 bp, while 40% of the hypomethylated lncRNAs were 400-600 bp (Figure 2C).

To understand the distribution of differentially M6A methylated lncRNAs across chromosomes, we further detected the enrichment level of M6A methylated lncRNAs on each chromosome. This revealed that hypermethylated lncRNAs were primarily located on chromosomes 2 (10.1%), 7 (11.6%), and 16 (11.8%) and hypomethylated lncRNAs were primarily located on chromosomes 1 (14.5%), 11 (12.7%), and 19 (8.5%) (Figure 2D).

Further analysis of the proportions of the differentially M6A methylated lncRNAs showed that 42.3% of the differentially M6A methylated lncRNAs were in the exon sense-overlapping group (Figure 2E). Among the hypermethylated lncRNAs, those in the intergenic group showed the highest fold change, whereas among the hypomethylated lncRNAs, those in the intronic antisense group had the highest fold change (Figure 2F).

### Functional analysis of genes near differentially methylated lncRNAs

To reveal the role of differentially methylated lncRNAs in the occurrence and development of CRC, we performed GO and KEGG pathway analysis on the genes located near differentially methylated lncRNAs.

**Figure 3:**
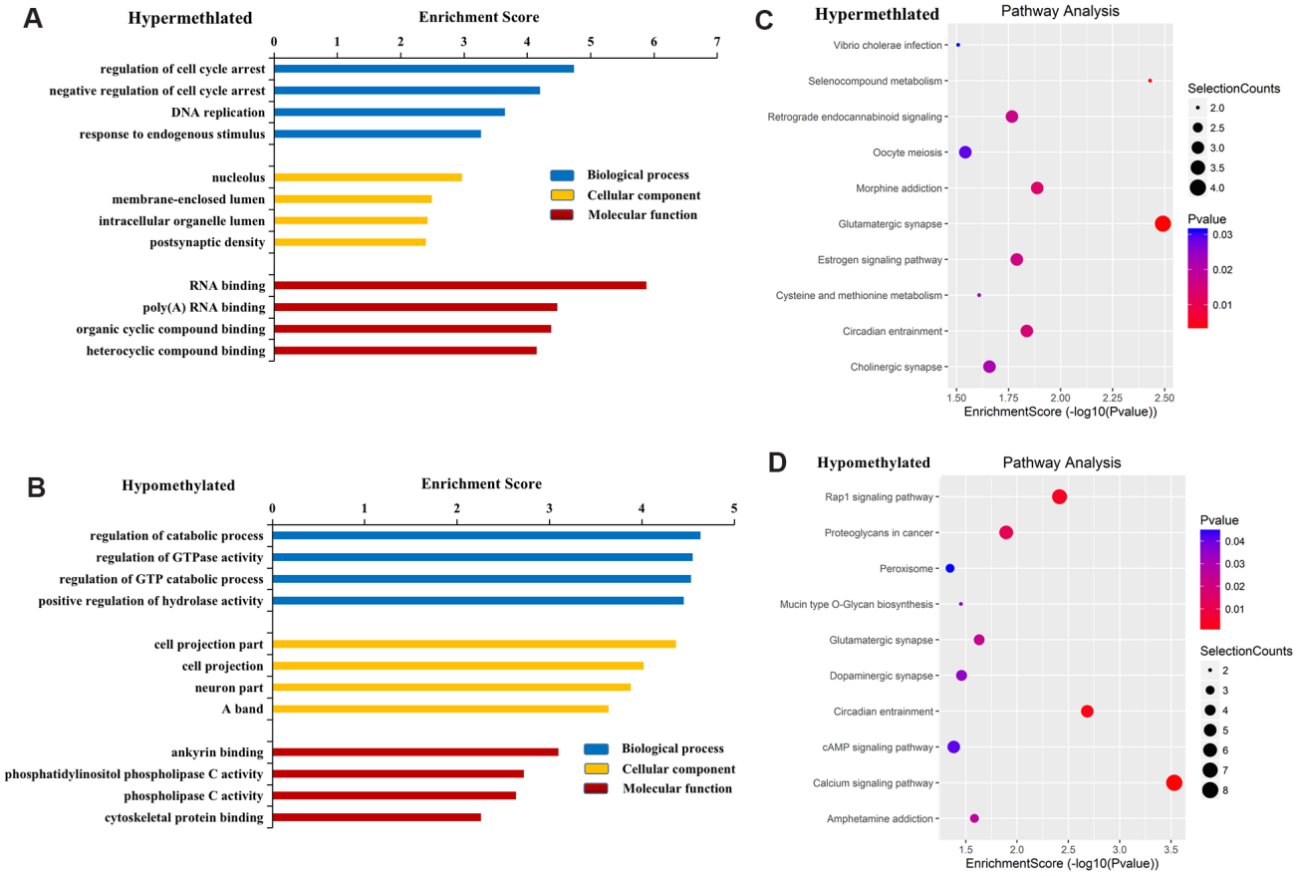
**Functional analysis of mRNAs located near differentially methylated lncRNAs.** GO enrichment analysis of genes near M6A hypermethylated (**A**) and hypomethylated (**B**) lncRNAs. GO enrichment analysis included BP analysis, CC analysis, and molecular function (MF) analysis. KEGG pathway analysis of genes near M6A hypermethylated (**C**) and hypomethylated (**D**) lncRNAs. p-values were calculated using DAVID.

GO results revealed that genes near hypermethylated lncRNAs primarily participate in the regulation of cell cycle arrest, DNA replication, and response to endogenous stimulus in the BP category. In terms of CC, genes are primarily involved in the nucleolus, membrane-enclosed lumen, intracellular organelle lumen, and postsynaptic density. In terms of MF, genes are primarily involved in RNA binding, poly (A) RNA binding, and organic cyclic compound binding (Figure 3A). Genes near hypomethylated lncRNAs are primarily involved in the following BPs: regulation of catabolic process, regulation of GTPase activity, and positive regulation of hydrolase activity. In terms of CC, genes are associated with cell projections and neurons. In terms of MF, genes primarily participate in ankyrin binding, phosphatidylinositol phospholipase C activity, and phospholipase C activity (Figure 3B).

KEGG pathway analysis revealed the four most important signaling pathways associated with genes near hypermethylated lncRNAs to be glutamatergic synapse, selenocompound metabolism, morphine addiction, and circadian entrainment (Figure 3C). For genes near hypomethylated lncRNAs, the four most important signaling pathways were the calcium signaling pathway, circadian entrainment, Rap1 signaling pathway, and dopaminergic synapse (Figure 3D).

These results suggest that M6A modified lncRNAs may affect the occurrence and development of CRC through biological processes, cell composition, MF, and signaling pathways.

### Overview of differentially expressed lncRNAs in CRC

**Figure 4:**
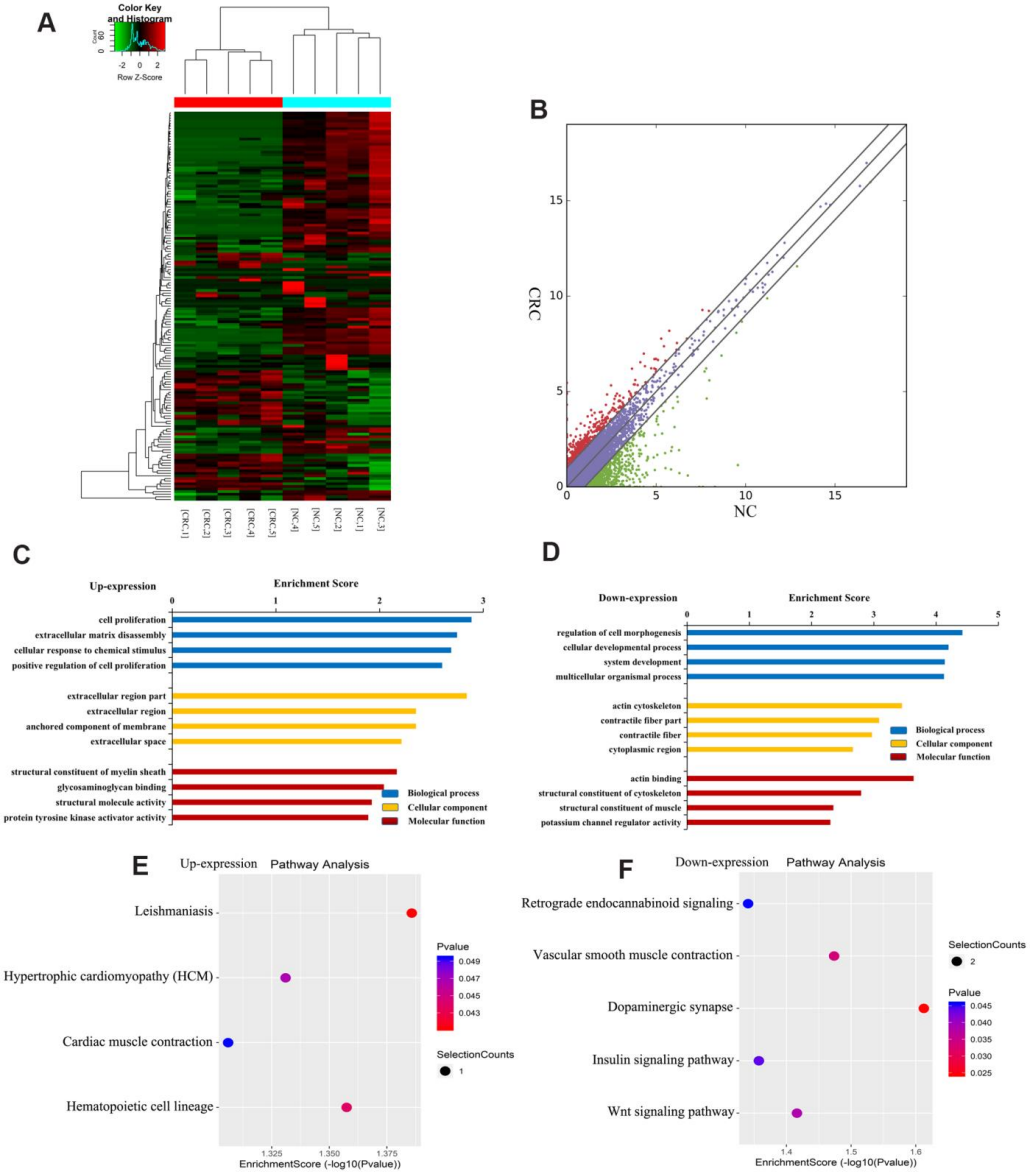
**Identification of differentially expressed lncRNAs in CRC.** (**A**) Heat map of RNA sequencing data from CRC and tumor-adjacent normal tissues. Rows: lncRNAs; columns: CRC and tumor-adjacent normal tissue samples. Red, green, and black indicate the upregulation, unchanged expression, and downregulation of lncRNAs, respectively. (**B**) Scatter plot of RNA sequencing data. GO enrichment analysis of genes near upregulated (**C**) and downregulated (**D**) lncRNAs. GO enrichment analysis included biological process (BP) analysis, cellular component (CC) analysis, and molecular function (MF) analysis. KEGG pathway analysis of genes near upregulated (**E**) and downregulated (**F**) lncRNAs. p-values were calculated using DAVID.

Figure 4A shows a heatmap of differentially expressed lncRNAs, indicating that these lncRNAs have different expression patterns in the two groups. There were 163 differentially expressed lncRNAs in CRC, of which 44 were upregulated and 119 were downregulated (Figure 4B). The top ten upregulated or downregulated lncRNAs are listed in Table 2.

**Table 2:** Top ten upregulated or downregulated expression lncRNAs.

**chrom**	**txstart**	**txend**	**transcript_id**	**Fold change**	**Regulation**
chr5	133853500	133853785	ENST00000464317	inf	up
chr15	70319263	70636611	ENST00000560903	831937553.1	up
chr17	70319263	70636611	ENST00000457958	20.9	up
chr4	83343716	83351294	uc.144-	8.6	up
chr1	121484056	121485434	TCONS_00001651	5.6	up
chrX	45707327	45710920	ENST00000414772	5.0	up
chr21	44232379	44237997	TCONS_00029036	4.6	up
chr1	61005920	61106163	ENST00000439156	4.5	up
chr8	128220110	128231333	ENST00000500112	4.4	up
chr2	43254991	43266686	ENST00000422351	4.1	up
chr2	54683421	54896812	uc021vhp.1	170654119.5	down
chr8	24808467	24814624	ENST00000221169	528.6	down
chr21	17442841	17999716	ENST00000458468	243.2	down
chr15	99676527	99791428	ENST00000564527	195.0	down
chr4	119809995	119982402	uc021xrd.1	119.2	down
chrX	31115793	33357558	uc022buq.1	62.7	down
chr3	64501332	64997143	ENST00000460833	52.2	down
chr9	71150805	71155783	uc004ags.1	49.6	down
chr14	103673867	103675204	TCONS_l2_00007928	49.3	down
chr5	148786251	148812399	ENST00000505254	46.7	down

To further understand the effects of genes near lncRNAs, we identified the genes near differentially expressed lncRNAs and analyzed their GO enrichment and KEGG pathways. GO analysis revealed that genes near upregulated lncRNAs were significantly enriched in cell proliferation, extracellular region, and glycosaminoglycan binding (Figure 4C). Genes near downregulated lncRNAs were significantly involved in the cell developmental process, actin cytoskeleton, and actin binding (Figure 4D).

KEGG pathway analysis revealed that genes near upregulated lncRNAs were primarily involved in leishmaniasis, myocardial contraction, hypertrophic cardiomyopathy, hematopoietic cell pedigree, etc. (Figure 4E). Genes near downregulated lncRNAs were mainly involved in retrograde endocannabinoid signaling, vascular smooth muscle contraction, dopaminergic synapse, insulin signaling, and Wnt signaling (Figure 4F). Together, these results indicate that genes near differentially expressed lncRNAs may be related to CRC.

**Figure 5:**
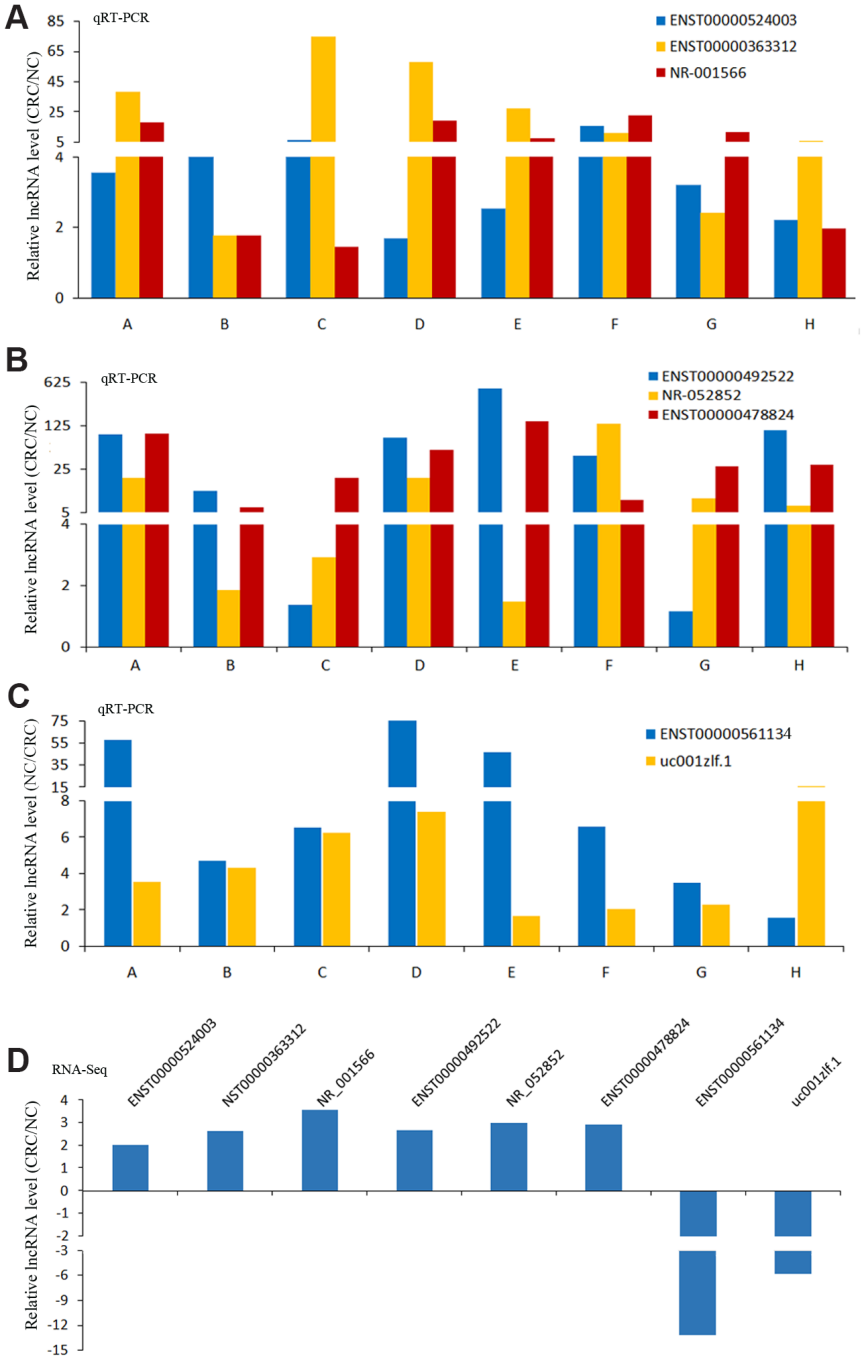
**The results of qRT-PCR and RNA-Seq for lncRNAs.** The results of qRT-PCR for lncRNAs ENST00000524003, ENST00000363312 and NR-001566 (**A**), lncRNAs ENST00000492522, NR-052852 and ENST00000478824 (**B**), lncRNAs ENST00000561134 and uc001zlf.1 (**C**). The RNA-Seq results of these lncRNAs (**D**).

In these differentially expressed lncRNAs, we used qRT-PCR to detect the expression of 8 lncRNAs in CRC and NC tissues. The results of qRT-PCR showed same expression trend with the RNA-Seq results (Figure 5). LncRNAs (ENST00000524003, ENST00000363312, NR-001566, ENST00000492522, NR-052852, ENST00000478824) were upregulated in CRC, and lncRNAs (ENST00000561134, uc001zlf.1) were downregulated (Figure 5).

### Association of M6A methylation and expression of lncRNAs

**Figure 6:**
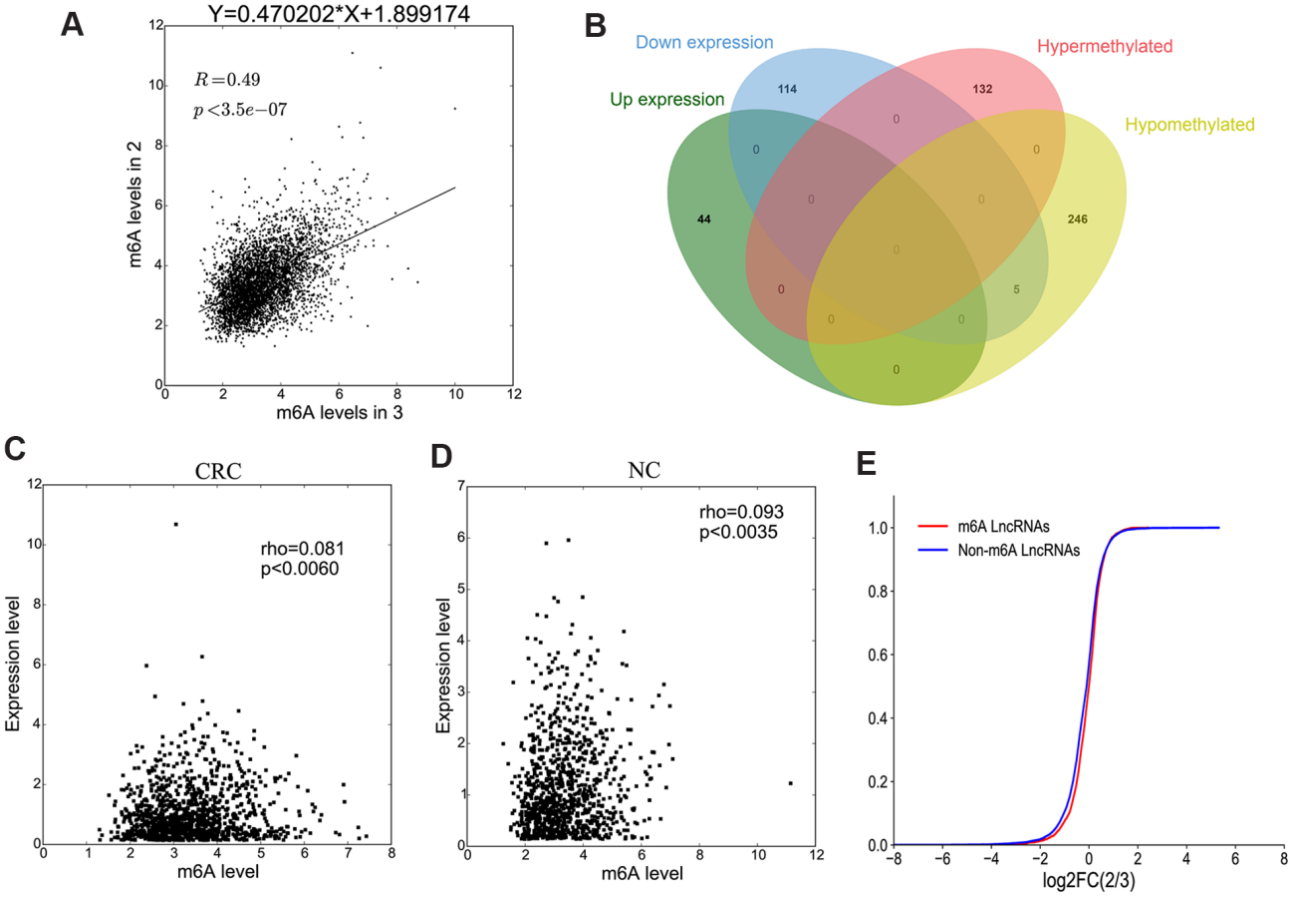
**The association between lncRNA M6A methylation and expression.** (**A**) The scatter plot shows the correlation of lncRNA M6A methylation between in CRC and NC. (**B**) Venn diagram showing the relationship between M6A modification and expression. (**C**, **D**) The scatter plot shows the correlation between lncRNA M6A methylation level and expression level in CRC and NC. (**E**) The lncRNA cumulative curve.

Methylation sequencing data showed that the level of lncRNAs methylation in CRC group was significantly higher than that in NC group (Figure 6A). Here we further analyzed the methylation correlation between the two groups. The correlation graph showed that the M6A level of lncRNAs in CRC group was positively correlated with the M6A level of lncRNAs in NC group (Spearman-related Rho=0.49, p < 0.05).

Combining the results of methylation sequencing and RNA sequencing, we found that 132 lncRNAs were hypermethylated, but of which was not differentially expressed lncRNAs. 251 lncRNAs were hypomethylated, among them, 5 lncRNAs were down-expressed (Figure 6B). To analyze the correlation between lncRNA methylation level and expression level, a correlation graph was constructed using the fold enrichment of lncRNA M6A methylation and expression value in terms of FPKM. The results indicated that there was a statistically significant positive correlation between methylation and expression levels of lncRNAs in CRC and NC (Figure 6C, 6D).

To further analyze whether M6A methylation affects lncRNA expression, we divided all expressed lncRNAs into M6A lncRNAs and non-M6A lncRNAs, calculated the log two fold change (log2FC) values of these lncRNAs, and generated a cumulative curve. The result revealed that the proportion of lncRNAs not modified by M6A was larger than that of lncRNAs modified by M6A, especially in terms of the log2FC of the lncRNA FPKM value between 0 and 2 (Figure 6E).

### Construction of lncRNA-miRNA-mRNA and lncRNA-mRNA networks in CRC

**Figure 7:**
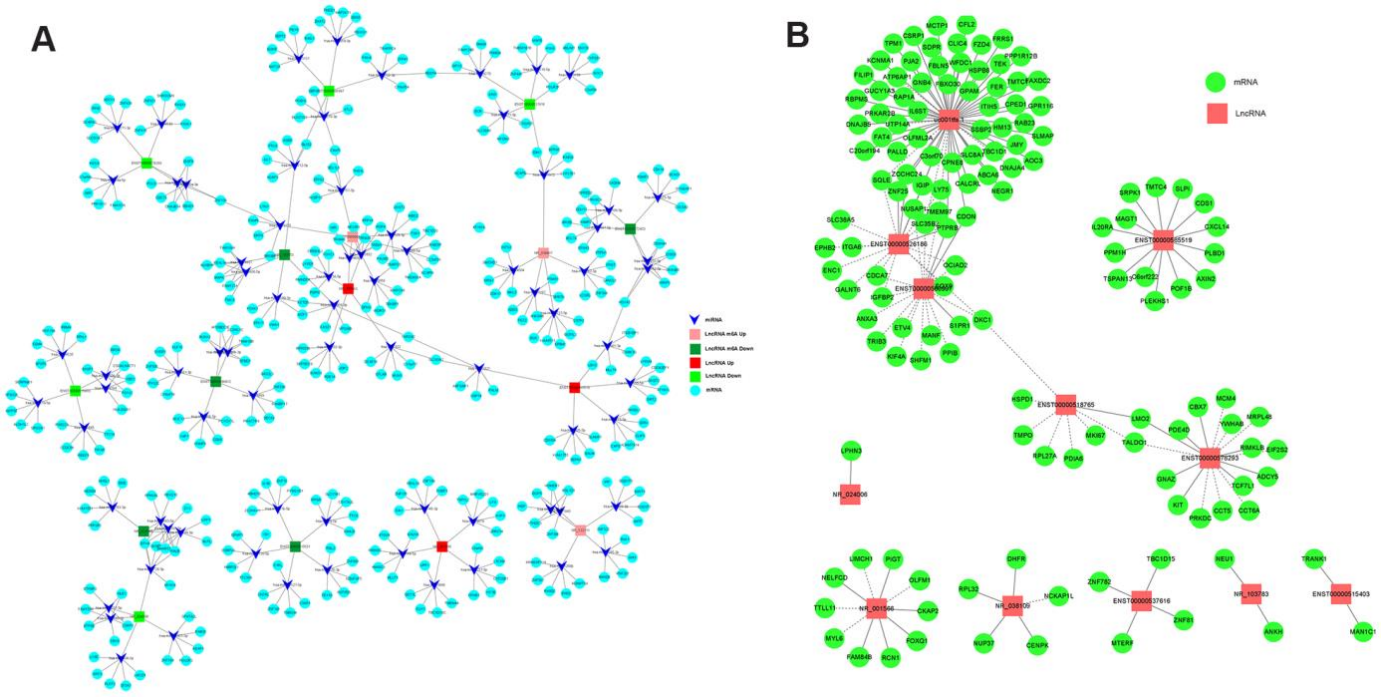
**The networks of lncRNA-miRNA-mRNA and lncRNA-mRNA regulation in CRC.** (**A**) The network of lncRNA-miRNA-mRNA regulation in CRC. (**B**) The network of lncRNA-mRNA regulation in CRC.

To explore the mRNAs regulated by lncRNAs, we screened eight lncRNAs with fold changes >7 out of 267 differentially methylated lncRNAs (Table 3) and eight lncRNAs with fold changes > 2.5 out of 158 differentially expressed lncRNAs (Table 3), all of which were associated with CRC. A ceRNA network was constructed by lncRNA-miRNA-mRNA association analysis. The network consisted of the top five miRNAs combined with a screened lncRNA and the top five mRNAs bound to the miRNAs, including 16 lncRNAs, 80 miRNAs, and 400 mRNAs. From this ceRNA network, it is clear that lncRNAs regulate to miRNAs and mRNAs. For example, NR_103783 could act as a sponge for hsa-mir-3652/hsa-mir-4430 to affect TRIM40 expression, and hsa-miR-138-5p may be sponged to regulate FOXC1 expression (Figure 7A).

**Table 3:** LncRNAs screened for lncRNA-miRNA-mRNA analysis.

**transcript_id**	**Foldchange**	**Regulation**	**Disease.Name**
ENST00000515531	50.20	hypomethylated	colon cancer
ENST00000433644	12.21	hypermethylated	CRC
ENST00000572453	11.04	hypomethylated	CRC
NR_122111	8.911	hypermethylated	CRC
ENST00000498432	10.44	hypomethylated	CRC
NR_038407	7.37	hypermethylated	CRC
NR_103553	8.88	hypomethylated	CRC
NR_026788	8.45	hypomethylated	CRC
ENST00000566997	-Inf	Down-expression	CRC
ENST00000578293	9.72	Down-expression	CRC
ENST00000537616	5.45	Down-expression	CRC
ENST00000515403	4.27	Down-expression	CRC
NR_001566	3.57	Up-expression	colon cancer
ENST00000565519	3.39	Up-expression	CRC
NR_024006	3.07	Down-expression	CRC
NR_103783	2.79	Up-expression	CRC

Next, to investigate the regulatory relationship between lncRNAs and mRNAs in CRC cells, we screened 12 differentially expressed lncRNAs that were related to CRC (Table 4) and analyzed the lncRNA-mRNA regulatory network. The network consisted of 12 lncRNAs and 158 mRNAs. The lncRNA with the largest number of chains was uc001tfa.1, which has 61 edges. The lncRNA uc001tfa.1 can positively regulate the expression of ITIH5 and RAP1 and negatively regulate the expression of SQLE, TMEM9, and USAP1 (Figure 7B). These two networks provide a deeper understanding of the functional role of lncRNAs in CRC.

**Table 4:** LncRNAs screened for regulatory analysis of LncRNA-mRNA.

**gene_id**	**transcript_id**	**Fold.change**	**Regulation**	**Disease.Name**
ENSG00000253123	ENST00000518765	Inf	down-expression	CRC
ENSG00000254510	ENST00000526186	Inf	down-expression	CRC
ENSG00000260182	ENST00000566997	Inf	down-expression	CRC
RMST	uc001tfa.1	Inf	down-expression	colon cancer
ENSG00000196295	ENST00000578293	9.7	down-expression	CRC
ENSG00000257027	ENST00000537616	5.45	down-expression	CRC
ENSG00000248596	ENST00000515403	4.27	down-expression	CRC
TERC	NR_001566	3.57	up-expression	colon cancer
ENSG00000261572	ENST00000565519	3.39	up-expression	CRC
LINC00950	NR_024006	3.07	down-expression	CRC
BLACAT1	NR_103783	2.79	up-expression	CRC
SNHG16	NR_038109	2.18	up-expression	CRC

## DISCUSSION

In this study, we found significant differences in M6A methylation and expression of lncRNAs between cancer tissues and tumor-adjacent normal tissues. GO and KEGG analyses indicated that genes near differentially methylated or expressed lncRNAs were involved in important biological pathways and signaling pathways, which were related to the occurrence and development of CRC. The correlation graph revealed a positive correlation between the methylation and expression levels of lncRNAs in CRC and NC. The cumulative curve indicated that the proportion of lncRNAs not modified by M6A was greater than that of lncRNAs modified by M6A, suggesting that methylation downregulates lncRNA expression. A ceRNA and lncRNA-mRNA expression-regulation network revealed a regulatory relationship between lncRNAs, miRNAs, and mRNAs. This study comprehensively profiled M6A modification and expression patterns of lncRNAs in human CRC to provide a deeper understanding of the functional role of lncRNAs in CRC and new ideas and directions for the diagnosis and treatment of CRC in the future.

KEGG and GO analyses were used to functionally profile the genes near differentially methylated lncRNAs to determine the role of M6A modified lncRNAs in CRC. Our results showed that genes near differentially methylated lncRNAs participate in the biological processes of cell cycle arrest and catabolism; are expressed in cellular components, such as nucleolus, cell cavity, and cell projection; and participate in other molecular functions, such as RNA binding, anchor protein binding, and phosphatidylinositol phospholipase C activity. In addition, genes proximal to differentially methylated lncRNAs were also found to be involved in the regulation of glutamatergic synapses, selenium compound metabolism, and calcium signaling pathways. Cell cycle arrest can provide an opportunity for tumor cells to repair their damaged DNA [27]. The progress of glutamate synaptic input drives tumors in glioma cells [28]. Selenium is a trace element with important benefits for both humans and animals. Selenium compounds exhibit good chemopreventive and chemotherapeutic effects in cancer treatment [29–30]. This indicates that M6A modified lncRNAs affect the occurrence and development of CRC through biological processes, cell composition, and molecular function signaling pathways.

LncRNA NR-001566 (lncRNA TERC) is a component of telomerase RNA, and its 11-base template region is considered as an ideal target for direct enzyme inhibition of telomerase activity [31]. LncRNA ENST00000500112 (LncRNA CCAT1), which was upregulated in CRC and esophageal cancer, by adjusting the expression of HOXB13 and SPRY4 affect cell proliferation and migration in esophageal squamous carcinoma [32, 33]. LncRNA ENST00000524003 (lncRNA OTUD6B-AS1) was downregulated in renal clear cells and inhibited the migration and invasion of cancer cells by inhibiting the activity of Wnt/ catenin pathway and epithelial-mesenchymal transformation [34]. In this study, we found that lncRNA TERC, lncRNA CCAT1 and lncRNA OTUD6B-AS1 were upregulated in CRC. The expression trend of lncRNA OTUD6B-AS1 in CRC and renal cell carcinoma is inconsistent, which may be related to tissue specificity and functional differences.

The relationship between lncRNA methylation and expression has always been a concern, but there is not yet a clear conclusion regarding this relationship. In our study, there was a positive correlation between methylation and expression levels of lncRNAs in CRC and NC. The cumulative curve revealed that the proportion of lncRNAs not modified by M6A was greater than that of lncRNAs modified by M6A, suggesting that methylation downregulates lncRNA expression. Previously, no comprehensive study has demonstrated the correlation between lncRNA methylation and expression. However, some studies on individual lncrRNAs have shown this correlation. Some studies have suggested that M6A methylation is positively correlated with lncRNA expression. M6A methylation was thought to be involved in the upregulation of lncRNA RP11 by increasing its nuclear accumulation [35]. M6A methylation improves the stability and level of RHPN1-AS1 by reducing RNA degradation [36]. However, other studies have found M6A levels to negatively correlate with lncRNA expression. For example, when the M6A methylation level of XIST was significantly decreased, the expression level of XIST was significantly increased [25].

In this study, we constructed a ceRNA and lncRNA-mRNA expression-regulation network to analyze the regulatory relationship between lncRNAs and miRNAs. From the two network diagrams, it can be seen that NR-103783 can serve as a sponge for hsa-mir-3652/hsa-mir-4430 to affect TRIM40 expression and for hsa-miR-138-5p to regulate FOXC1 expression. The lncRNA uc001tfa.1 can positively regulate the expression of ITIH5 and RAP1 and negatively regulate the expression of SQLE, TMEM9, and USAP1. The target genes of lncRNAs are related to the occurrence and development of tumors. TRIM40 has been reported to inhibit nuclear factor-kappaB (NF-κB) activity and prevent inflammation from causing cancer in the gastrointestinal tract [37]. High FOXC1 expression is closely related to metastasis, recurrence, and decreased survival rates in CRC [38]. ITIH5 is considered to be associated with extracellular matrix stability and may therefore play a key role in inhibiting tumor progression [39]. RAP1A promotes CRC development through the PTEN/FOXO3/CCND1 signaling pathway [40]. SQlE is a breast cancer oncogene [41]. TMEM9, through vacuolar-ATPase-activated Wnt/β-catenin signaling, promotes intestinal tumorigenesis [42]. Silencing of NUSAP1 inhibits cell proliferation, migration, and invasion by inhibiting DNMT1 expression in human CRC [43]. The abovementioned genes are only few of the tumor-related lncRNA target genes, and their functions require further investigation. However, from these genes, it is clear that the two network diagrams presented herein can both explain the regulatory relationship between lncRNAs and mRNAs and be of great significance to further understand the role of lncRNAs in the occurrence and development of CRC. This could provide a theoretical basis for the occurrence, diagnosis, and treatment of colon cancer at the transcriptional and post-transcriptional levels.

## MATERIALS AND METHODS

### Tissue samples

Tumor and tumor-adjacent normal tissue samples were collected from five patients with CRC who underwent surgical resection at the first affiliated Hospital of Chengdu Medical College. For tumor tissue samples, colorectal tissue samples with a diameter of approximately 0.5-1.0 cm were collected from the parenchyma tissue of patients with CRC. For non-tumor tissue samples, colorectal tissue samples with a diameter of approximately 0.5 cm-1.0 cm were collected from tissues 5cm away from the parenchyma of patients with CRC. Tissue samples were rinsed with normal saline and cryopreserved with liquid nitrogen for subsequent RNA isolation. Patients did not receive chemotherapy or radiotherapy prior to the operation and signed a written informed consent form. This study was approved by the Ethics Review Committee of the First Affiliated Hospital of Chengdu Medical College.

### RNA library preparation and sequencing

High-throughput RNA sequencing was performed by Cloud-Seq Biotech (Shanghai, China). RNA was extracted using TRIzol Reagent (Invitrogen, Carlsbad, CA, USA) according to the manufacturer's instructions. The NEBNext rRNA Depletion Kit (New England Biolabs, Inc., MA, USA) was used to remove ribosomal RNA from the total RNA. RNA concentration was determined using a NanoDrop ND-1000 spectrometer (Thermo, Waltham, MA, USA), and RNA integrity was detected by denaturing gel electrophoresis. RNA is considered pure when the value of NA OD260/OD280 falls between 1.8-2.1. RNA libraries were constructed using the NEBNext®Ultra™ II Directional RNA Library Prep Kit (New England Biolabs, Inc., MA, USA) according to the manufacturer’s instructions. Libraries underwent quality control and were quantified using a BioAnalyzer 2100 system (Agilent Technologies, Inc., CA, USA). Libraries were sequenced on an Illumina HiSeq instrument, with 150 bp paired-end reads.

### MeRIP (methylated RNA immunoprecipitation) library preparation and sequencing

Total RNA was extracted as described above. M6A RNA-Seq was performed by Cloud-seq Biotech Inc. (Shanghai, China). Briefly, M6A RNA immunoprecipitation was performed using the GenSeqTM M6A-MeRIP Kit (GenSeq Inc., China), according to the manufacturer’s instructions. Both the input samples without immunoprecipitation and the M6A IP samples were used to generate libraries for RNA sequencing using the NEBNext®Ultra II Directional RNA Library Prep Kit (New England Biolabs, Inc., MA, USA).

Library quality was evaluated using a BioAnalyzer 2100 system (Agilent Technologies, Inc., CA, USA). Libraries were sequenced on an Illumina HiSeq instrument, with 150 bp paired-end reads.

### Reverse-transcription quantitative polymerase chain reaction validation of RNA sequencing data

The lncRNAs (ENST00000524003, ENST00000363312, ENST00000478824, ENST00000492522, ENST00000561134, NR-052852, NR-001566, uc001zlf.1), which were fold change > 2 were selected to validate the accuracy of RNA sequencing via the reverse-transcription quantitative polymerase chain reaction (qRT-PCR). Primers were designed based on cDNA sequence (Table 5) using Primer Premier 5 and synthesized by Shanghai Genechem Co.,Ltd (Shanghai, China). Total RNA was extracted from tissues (8 pairs of CRC and NC tissues) using Tatal RNA Extraction KIT (Solarbio, Beijing, China) according to the manufacturer’s instructions. The concentration and purity of the isolated RNA was determined using Nanodrop 2000C microspectrophotometer (Thermo Scientific, New York, USA) and reversing transcription was performed according to the PrimeScript RT reagent kit with gDNA Eraser (Takara Biotechnology Co., Ltd, Beijing, China). RT-qPCR was performed using TB Green™ Premix Ex Taq™ II (Takara Biotechnology Co., Ltd, Beijing, China). For quantitative results, expression of lncRNA was expressed as fold change using the 2−ΔΔCt method and processed by SPSS19.0 with a one-way analysis of variance.

**Table 5:** The primers of lncRNAs for qRT-PCR detection.

**LncRNA**	**Primer sequence**	**Product size (bp)**
ENST00000524003	ATTCCCTGCTTCAGAGGACCTC	212
	CGCGCATAGGTGTTTAAGCTCC	
ENST00000363312	GTCTAACCCTAACTGAGAAGGG	122
	CTCTAGAATGAACGGTGGAAGG	
NR-001566	AAGAGTTGGGCTCTGTCAGCCG	126
	CACGTCCCACAGCTCAGGGAAT	
ENST00000492522	ACCTGCTGTGAAGCCTTGAAAAG	195
	GTTTGCATGCCAGTGAACATCTG	
NR-052852	ATGGGCAGCCAGAGCTCCAA	174
	GGCTGCCTCATCTGTTCCGT	
ENST00000478824	CAACTCACCTTCATGCTACATCT	127
	CCCACTATGGACAGTTTCCATTC	
ENST00000561134	AGAGACTATAGCCCAGGGCTCT	98
	GTCCCAGTTCCCAGTGGAAGAA	
uc001zlf.1	GGCCTATACTCTAGTTAGCTCTG	194
	CATAGGCTTCCCAATGACTGAA	

### Bio-informatic analysis of LncRNA targets and associated pathways

After sequencing, Q30 was used for quality control and cutadapt [44] (v1.9.3) was used to remove 3’ adaptors and low-quality reads to obtain clean high-quality reads. High-quality reads were aligned to the human reference genome (UCSC HG19) using Hisat2 [45] (v2.0.4). Then, guided by the Ensembl gtf gene annotation file, the FPKM [46] (Fragments per kilobase of exon per million fragments mapped) values of transcript-level lncRNAs and gene-level mRNAs were obtained using cuffdiff (part of v2.2.1). These values were used to form the expression profile of lncRNAs and mRNAs, and multiple changes and p-values between the two groups of samples were calculated to identify differentially expressed lncRNAs and mRNAs. MACS [47] was used to identify methylated sites in each sample. Differentially methylated sites were identified by diffReps [48]. We wrote a program to filter the peaks on lncRNA exons.

LncRNAs are non-protein coding RNAs, but they can regulate target gene expression. Therefore, the function of lncRNAs can be characterized by the function of their target genes [49, 50]. The target genes of lncRNA can be divided into two types, cis-targets and trans-targets [49]. The transcribed genes within a 10 kbp window upstream or downstream of lncRNAs location were considered as cis-target genes [50]. When the expression quantity correlation coefficient of a lncRNA and its corresponding target mRNA was p ≥ 0.9, it was considered to be a potential trans-target [49].

Annotation, visualization, and integrated discovery databases were used for gene ontology (GO) and pathway enrichment analyses. GO consists of three components: cell component (CC), molecular function (MF), and biological process (BP). The p-value indicates the significance of GO term enrichment. Pathway enrichment analysis is the functional analysis of genes mapped to specific pathways in the Kyoto Encyclopedia of Genes and Genomes (KEGG). The Fisher p-value indicates the importance of the path related to the condition.

We first screened lncRNAs related to CRC from the differentially methylated or expressed lncRNAs; then, we screened the lncRNAs with a methylation fold change >7 and the lncRNAs with an expression fold change >2.5 from the lncRNAs that were related to CRC. The miRNA-target gene prediction software based on Miranda [51] and TargetScan [52] was used to predict miRNAs and mRNAs which were combined for the screened lncRNAs. The ceRNA network was plotted using Cytoscape [53] (v3.7.1). The Pearson correlation coefficient of expression level between lncRNAs and mRNAs [54] was calculated, with a threshold value of 0.95, and a coding and non-coding co-expression (CNC) network was plotted using Cytoscape [53] (v3.7.1).

### Ethics approval and consent to participate

This study was approved by the Ethics Review Committee of the First Affiliated Hospital of Chengdu Medical College (permit number: CYYFYEC2019002).

### Data availability

If you need the raw high-throughput M6A and lncRNAs sequencing generated during the current study, you can ask the corresponding author by E-mail.
